# Exploring DrugCentral: from molecular structures to clinical effects

**DOI:** 10.1007/s10822-023-00529-x

**Published:** 2023-09-14

**Authors:** Liliana Halip, Sorin Avram, Ramona Curpan, Ana Borota, Alina Bora, Cristian Bologa, Tudor I. Oprea

**Affiliations:** 1 Department of Computational Chemistry, “Coriolan Dragulescu” Institute of Chemistry, Timisoara, Romania; 2 Translational Informatics Division, Department of Internal Medicine, University of New Mexico School of Medicine, Albuquerque, NM USA; 3 Expert Systems Inc, San Diego, CA USA

**Keywords:** Drugs, Pharmacology, Databases, Bioactivity, Drug targets, Drug repurposing

## Abstract

DrugCentral, accessible at https://drugcentral.org, is an open-access online drug information repository. It covers over 4950 drugs, incorporating structural, physicochemical, and pharmacological details to support drug discovery, development, and repositioning. With around 20,000 bioactivity data points, manual curation enhances information from several major digital sources. Approximately 724 mechanism-of-action (MoA) targets offer updated drug target insights. The platform captures clinical data: over 14,300 on- and off-label uses, 27,000 contraindications, and around 340,000 adverse drug events from pharmacovigilance reports. DrugCentral encompasses information from molecular structures to marketed formulations, providing a comprehensive pharmaceutical reference. Users can easily navigate basic drug information and key features, making DrugCentral a versatile, unique resource. Furthermore, we present a use-case example where we utilize experimentally determined data from DrugCentral to support drug repurposing. A minimum activity threshold *t* should be considered against novel targets to repurpose a drug. Analyzing 1156 bioactivities for human MoA targets suggests a general threshold of 1 µM: *t* = 6 when expressed as − log[Activity(M)]). This applies to 87% of the drugs. Moreover, *t* can be refined empirically based on water solubility (S): *t* = 3 − logS, for logS < − 3. Alongside the drug repurposing classification scheme, which considers intellectual property rights, market exclusivity protections, and market accessibility, DrugCentral provides valuable data to prioritize candidates for drug repurposing programs efficiently.

## Introduction

**Fig. 1:**
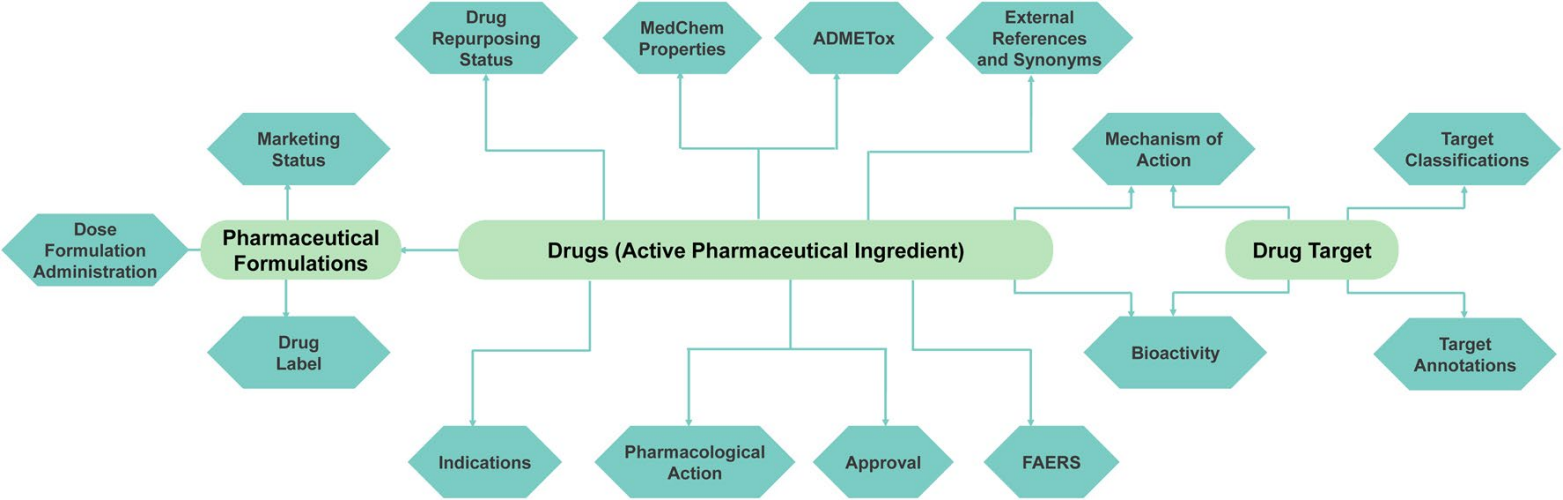
DrugCentral main entities and relations diagram

DrugCentral (http://drugcentral.org), established in 2016 [1] is an open-access drug compendium that connects the scientific basis of drug substances to approved pharmaceutical products for healthcare professionals. It encompasses drug structures and properties, regulatory details, bioactivity profiles, mechanism-of-action (MoA) targets, pharmacological actions, therapeutic applications and contraindications, adverse events, and drug formulations and products. Figure 1 demonstrates the main components and their links. Although some information is manually curated from scientific literature and drug labels, most data is aggregated from public online resources [1–4].

**Fig. 2:**
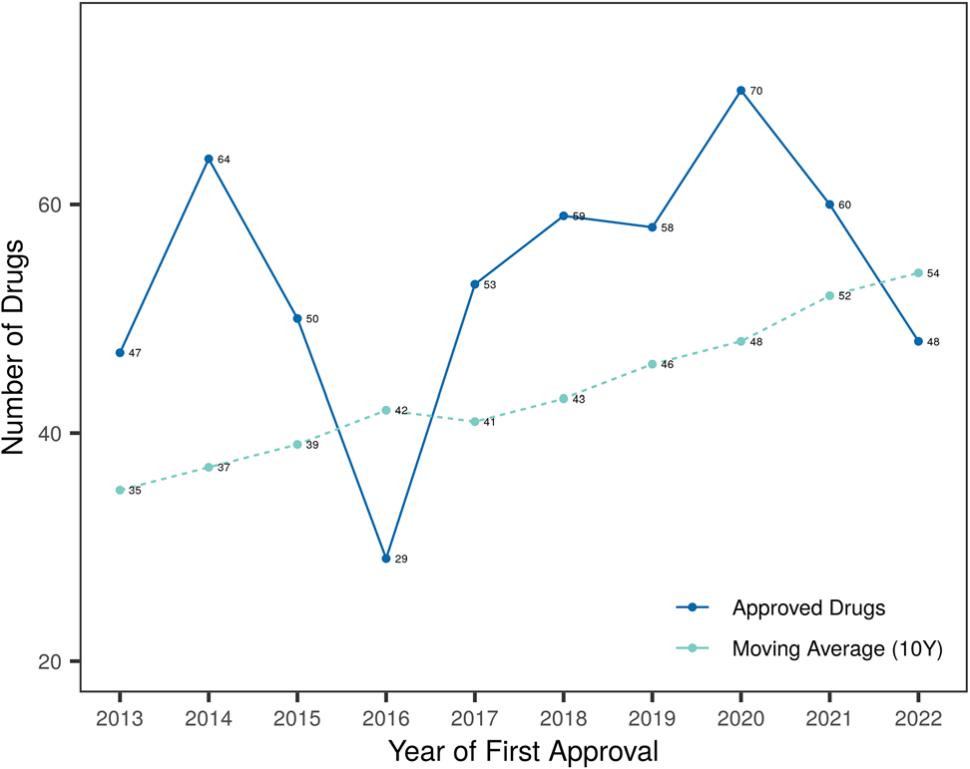
Number of first-time drug approvals per year (2013–2022) and moving average (1990–2022) for the past decade

DrugCentral tracks drug approvals from three major regulatory agencies: the U.S. Food and Drug Administration (FDA) [5], European Medicines Agency (EMA) [6], and Japan Pharmaceutical and Medical Devices Agency (PMDA) [7]. In the last decade, the number of drugs approved for the first time per year varied between 29 and 70. Overall, the moving average of the past 10 years indicates an increasing trend (Fig. 2).

Here we describe DrugCentral content (related to 4959 drugs) with various features and functionalities (based on the entire 2022 release) added during the past years [1–4]. We briefly discuss drug properties, targets, clinical effects, drug products, pharmaceutical formulations, and data accessibility.

## Drug properties

DrugCentral comprises 4959 active drug substances approved for human (4805) and veterinary use (396) [4], with properties computed or sourced externally. Various classification schemes, such as drug types, pharmacological classes, market availability, and patent coverage, enable users to select specific data sets. External identifiers link DrugCentral entries to complementary databases, creating a network of interconnected resources that provide users with a broad range of drug information.

### Types of drugs

DrugCentral categorizes drugs into distinct types: small molecules, peptides, biologics (antibodies, antibody–drug conjugates, proteins, fused-proteins, oligonucleotides, small interfering RNA), and others (coordination, dendrimer, inorganic and organometallic molecules, polymer and radiopharmaceuticals). The majority of entries are small organic molecules (82.8%, 4108 entries), while peptides (133 entries) and biologics (260 entries) constitute 7.9% of the total (Table 1). Since 2020, biologics have gained traction, making up one-third of new approvals (59 out of 178). DrugCentral data indicates that 40.3% of all approved biologics have orphan designations. Conversely, only 6.8% of small molecules and 18% of peptide drugs are approved for rare diseases (Table 1).

**Table 1:** A brief description of the types of drugs indexed by DrugCentral

Drug types	Small organic molecules	Peptides	Biologics	Other	Total
Number of drugs	4108	133	260	458	4959
Number of orphan drugs	278	24	105	27	434
Number of approved drugs since 2020	93	9	59	17	178

### Chemical structures and descriptors

Small-molecule and peptide drug chemical structures are accurately represented at the molecular level, with manual entry using depictions and information from sources like the WHO-INN [8]; United States Adopted Name, USAN [9]; and FDA drug labels [4]. Occasionally, information is verified through Chemical Abstract Service, CAS [9]. Drugs are standardized as follows: (i) Ionic salts have counterions removed and hydrated/solvated drug formulations have water/solvent removed (e.g., various atorvastatin calcium formulations are mapped to the single active ingredient, atorvastatin); (ii) Ester prodrugs are stored as-is (e.g., olmesartan medoxomil instead of its active metabolite, olmesartan); both enalapril and enalaprilat are indexed as enalapril is formulated both as a free acid and as the maleate salt of enalapril, the ethyl ester of enalaprilat [1].

Chemical structures of 4288 drugs have been manually entered and validated, with molecular weight (MW) spanning from 4 (Helium, a medically-used noble gas) to 22,125 a.m.u. (somatotropin, a 191-amino acid protein growth hormone). Several physicochemical properties relevant to drug analysis are available for small organic molecules, including the key properties used in the Lipinski rule of 5 (Ro5) criteria [10]: MW, number of rotatable bonds (RTB) [11], hydrogen bond donors/acceptors (HBD, HBA), and the calculated 1-octanol/water partition coefficient, CLOGP [12]—calculated using Biobyte software (http://www.biobyte.com/). Other properties encompass the number of rings and the topological polar surface area, TPSA [13]. DrugCentral enhances the chemical profiles of drugs, essential for understanding pharmacokinetics and toxicity, by calculating protonation constants with MOKA 3.0 software [14].

**Fig. 3:**
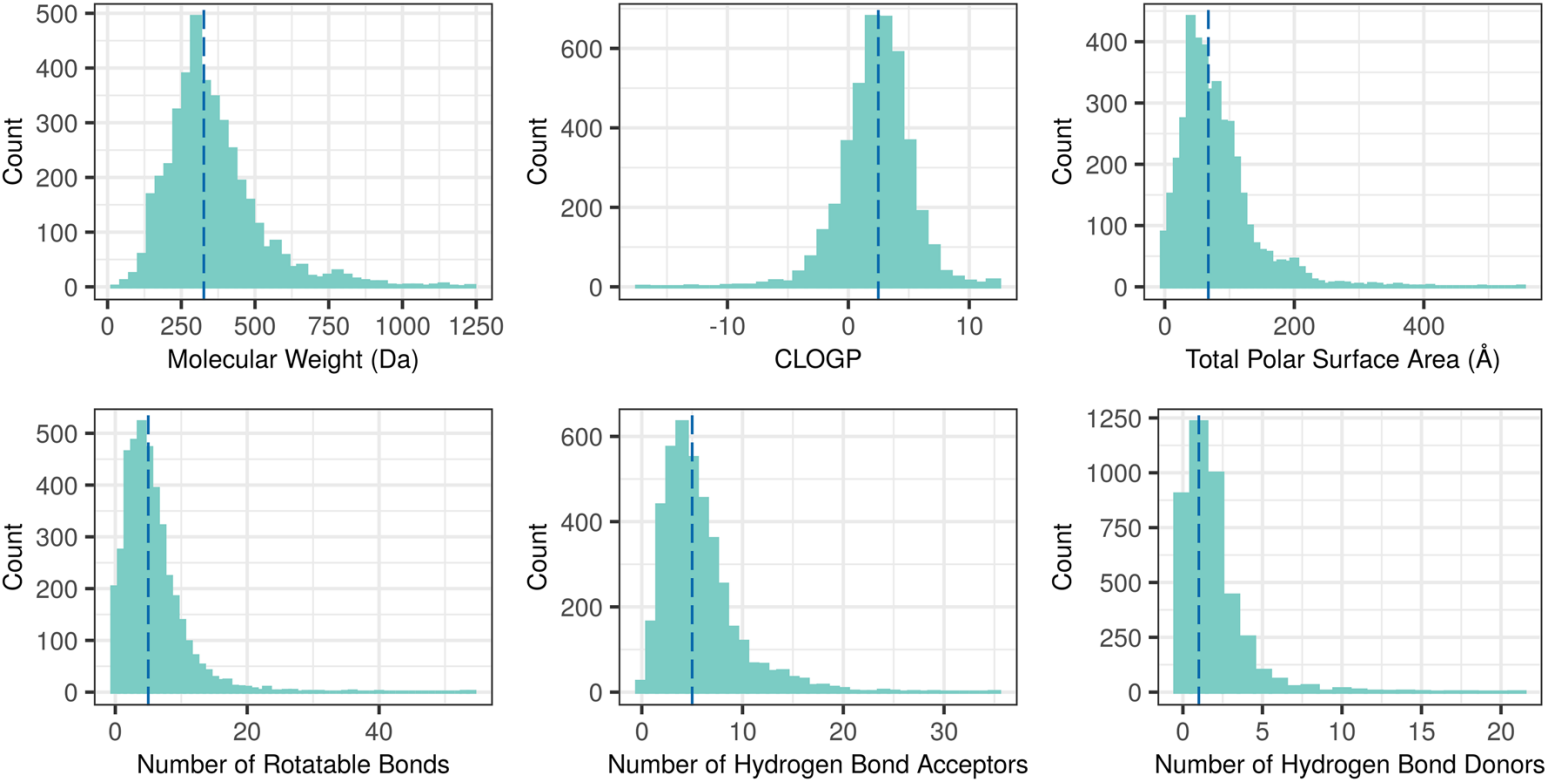
Calculated property distribution of DrugCentral small molecules

Figure 3 displays the distribution of six well-known properties calculated for 4092 small-molecule drugs (MW < 1250 a.m.u.). Median values are 326 for MW, 2.48 for CLOGP, 67.3 for TPSA, 5 for RTB, 5 for HBA, and 1 for HBD. Ninetieth percentile values for these physicochemical properties of drugs are as follows: MW ≤ 550, CLOGP ≤ 5.5, TPSA ≤ 145, RTB ≤ 10, HBA ≤ 10, HBD ≤ 4. The majority of these drugs (75%) are Ro5 compliant.

## Pharmacology and external links

### Pharmacokinetics

DrugCentral captures pharmacokinetics (absorption, distribution, metabolism, and excretion) using nine representative properties: BDDCS, biopharmaceutical drug disposition classification system [15]; the maximum recommended therapeutic daily dose, MRTD; clearance, Cl; half-life T_1/2_; the volume of distribution, Vd; absolute oral bioavailability, BA; the extent of metabolism, EoM; fraction unbound, fu; and water solubility, S [3]. Except for the last three, these properties are accessible for over 900 drugs each [3].

### Pharmacologic classes

DrugCentral provides a range of pharmacological classifications. The manual allocation of WHO INN [8] stems allows for categorization based on chemical and pharmacological attributes. Where possible, drugs are connected to the most recent versions of ChEBI ontology [16], Medical Subject Headings, MeSH terms [17], and the FDA Established Pharmacologic Class, EPC [18]. In DrugCentral, 4230 unique drugs are linked to 2721 pharmaco-chemical class codes, with the distribution displayed in Table 2.

**Table 2:** Pharmacological classes of drugs

Sources	ChEBI [16]	MeSH [17]	FDA [18]	WHO STEM [8]
Number of class codes	701	466	1131	424
Number of drugs	2230	3017	3229	2845

### Drug repositioning categories

DrugCentral implemented a multi-category drug repositioning scheme [19], capturing information on patent and market status. Drugs are categorized based on intellectual property rights, market exclusivity protections, and market accessibility: off-patent (OFP) for on-market drugs with expired patents/exclusivities; on-patent (ONP) for on-patent, on-market drugs with active patents/exclusivity rights; and off-market (OFM) for discontinued or withdrawn drugs. This classification aids the drug repurposing and repositioning community, with 279 drugs as ONP, 1038 as OFP, and 402 as OFM. Drug repositioning strategies should prioritize drugs based on their intellectual property landscape and marketing status in the following order: (i) OFP, (ii) OFM, and (iii) ONP [19].

### External identifiers

**Fig. 4:**
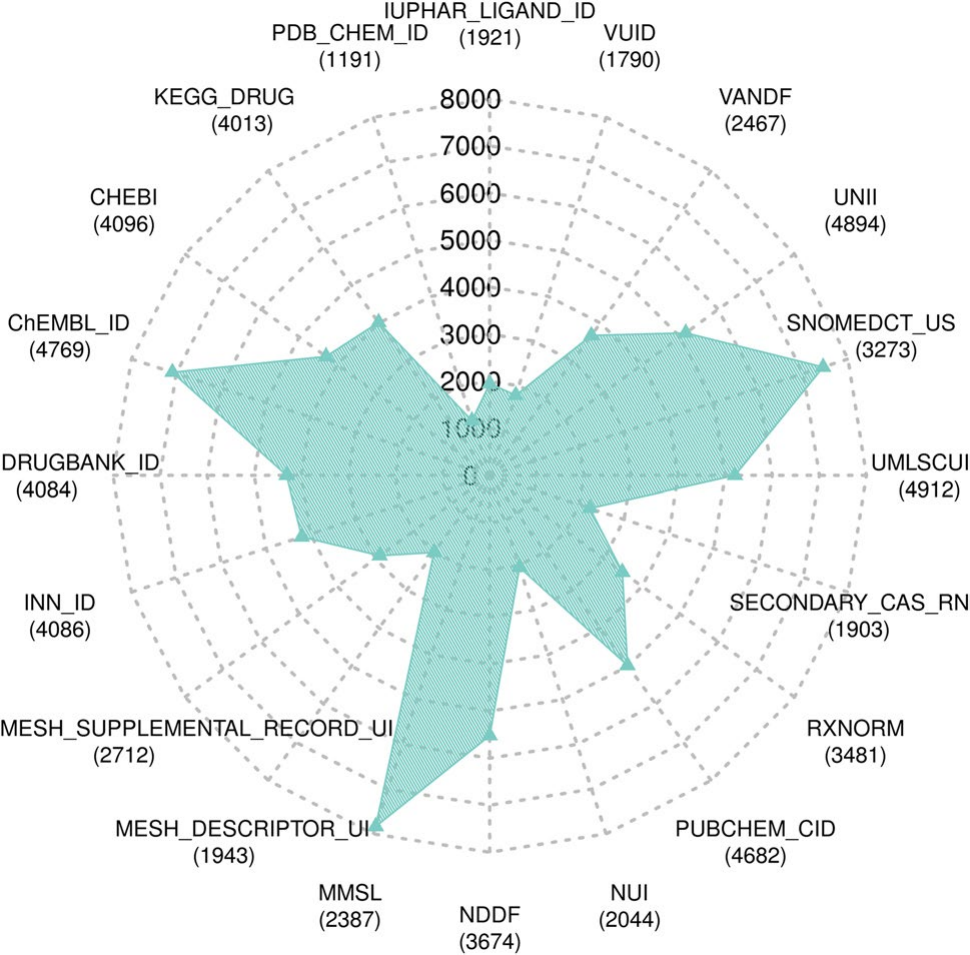
The number of identifiers from external sources, with the corresponding number of DrugCentral entries mapped to each identifier in parentheses, displayed as web plot. For example, 4769 drugs were mapped to approximately 7000 ChEMBL entries

DrugCentral utilizes external digital resources for swift access to complementary data. CAS registry numbers [20], WHO INN IDs, and KEGG identifiers [21] are manually curated, while twelve other identifiers are automatically assigned based on drug names, synonyms, and chemical structures (InChIKey). Figure 4 displays the current number of identifiers and corresponding drug count in DrugCentral.

## Drug targets and bioactivity data

DrugCentral captures quantitative bioactivity data for drug-target interactions when available. Comprehensive drug-target bioactivity profiles are compiled through automated extraction from multiple digital resources and enhanced by manual curation. This includes MoA (mechanism of action) targets, which are molecules (e.g., proteins, biopolymers, metabolites, metal atoms, drugs) that the drug (or its active metabolite) binds to for the intended therapeutic effect [22]. DrugCentral manually assigns MoA designations [23], offering a mechanistic understanding of drug actions related to human diseases and symptoms at the molecular level. MoA targets are manually annotated in DrugCentral using a curated list of external resources and expert curation. Drug labels and data reviewed by regulatory agencies are the primary sources for MoA target assignments. For approved drugs lacking this information in drug label data (e.g., pre-1990s approved drugs, some PMDA-approved drugs), MoA targets are critically assessed using scientific literature [23].

### Bioactivity data

DrugCentral contains 20,658 activity endpoints (drug-target pairs) for 2715 drugs across 3171 targets. Bioactivities are compiled from various sources: ChEMBLdb [24] (59.7%), WOMBAT-PK [25] (13.8%), DrugMatrix [26] (11.0%), IUPHAR/BPS Guide to Pharmacology [27] (6.1%), scientific literature (3.7%), PDSP [28] (3.6%), and drug labels (1.6%) (Table 3). Multiple types of bioactivity data are stored: dissociation constants (K_d_), and inhibition constants (K_i_) but also inhibitory concentrations 50% (IC_50_) and effective concentrations 50% (EC_50_) accounting for drug-target interactions. Most activity determinations consist of K_i_ (7871) and IC_50_ (6043) values, followed by K_d_ (4141) and EC_50_ (898) values. Since IC_50_ and EC_50_ generally depend on assay details (such as concentration and K_m_ of the substrate), users are advised to carefully check assay details when using bioactivity data to interpret in vivo effects. Among drug targets, 1795 (56.6%) are of human origin and interact with 2455 drugs.

**Table 3:** Summary of bioactivity data (MoA counts in parentheses)

Bioactivity source	Number of endpoints	Number of drugs	Number of targets with UniProt [29] IDs
ChEMBLdb [24]	12,328 (941)	1845 (659)	2506 (354)
WOMBAT-PK [25]	2845 (720)	913 (477)	599 (182)
DrugMatrix [26]	2255 (42)	344 (34)	97 (21)
IUPHAR [27]	1267 (206)	549 (174)	532 (138)
Scientific literature	763 (502)	389 (342)	412 (285)
PDSP [28]	734 (23)	182 (18)	69 (9)
Drug labels	326 (258)	218 (206)	214 (168)

### Target classes

**Fig. 5:**
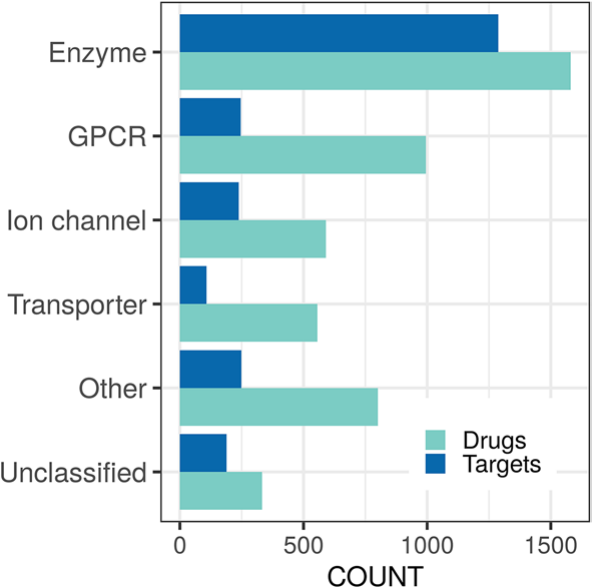
Count of targets and drugs according to target types

In DrugCentral’s bioactivity data, the largest target group are enzymes (53.5%; 15.6% are kinases), followed by G protein-coupled receptors—GPCRs (15.1%), and ion channels (11.1%). Most drugs (71%) target enzymes and GPCRs. The number of drugs exceeds the number of targets (Fig. 5), except for protein kinases, indicating a lack of selectivity among protein kinase inhibitors [19, 30, 31].

### Mechanism-of-action targets

DrugCentral indexes 724 mechanism-of-action (MoA) protein targets (598 of human origin) with referenced UniProt IDs for 1905 drugs. Most of the MoA activity data (81% of 3491 activities) are derived from ChEMBL, WOMBAT-PK, and scientific literature (Table 3). Enzymes (34.9%), GPCRs (15.7%), and ion channels (14.6%) make up the majority of targets (473 in total), accounting for the therapeutic effects of 1287 drugs (68%). This includes 1148 small organic molecules targeting 413 enzymes, GPCRs, and ion channels as MoA targets. Within the group of biologics, monoclonal antibodies (mAb) are the most represented (91 drugs), binding to 57 MoA targets. Most of these targets are cytokines, surface antigens, and membrane receptors. Between 12 and 17 new MoA targets have appeared yearly, with increasing trends of mAb and ADCs over small molecule drugs, according to the *Nature Reviews Drug Discovery* TargetWatch series (Table 4) [32–36]**.**

**Table 4:** Summary of novel drug targets published annually in the TargetWatch series

TargetWatch publications		2018 [32]	2019 [33]	2020 [34]	2021 [35]	2022 [36]
Novel targets		16	17	13	15	12
Drug types	Small molecules	6	5	5	3	5
	mAbs^a^ and ADCs^b^	7	4	6	10	5
	Other drug types	1	5	5	4	2

^a^Monoclonal antibodies; ^b^antibody–drug conjugates

### Target development levels

DrugCentral employs a knowledge-based classification scheme for targets, categorizing human proteins into four classes based on their target development level (TDL) [37]:

- Tclin—assigned to drug targets involved in the MoA of approved drugs.
- Tchem—annotates proteins known to bind small molecules with high affinity but not Tclin.
- Tbio—designates proteins with significant biological experimental data available.
- Tdark—refers to understudied proteins (covering ~ 29% of the human proteome).

Currently, DrugCentral has 709 Tclin and 485 Tchem targets, which are supplied (with supporting information) to the Target Central Resources Database (TCRD) [38] and further linked to the Pharos portal [38, 39].

## Drug uses and adverse events

DrugCentral indexes drug-disease information, including approved and off-label uses, contraindications, and adverse events. Regulatory agencies grant drug approval based on substantial evidence of safety and efficacy for specific clinical situations, as indicated in the drug label (on-label drug use or drug indication). However, once a drug is on the market, physicians (or healthcare providers) can prescribe it off-label to address clinical conditions or diseases that are not on the list of approved indications. Off-label usage often supplements existing therapies to treat diseases without approved treatment options. DrugCentral provides information on therapeutic uses (on-label and off-label) and contraindications for a comprehensive understanding of the drug’s applications. Furthermore, pharmacovigilance data processed from the FDA’s Adverse Event Reporting System (FAERS) [40] is integrated into DrugCentral entries, providing details on adverse drug reactions. Currently, DrugCentral includes therapeutic use and adverse event information for 3278 drugs.

### Indications, contraindications, and off-label uses

In DrugCentral, indications, contraindications, and off-label uses have been extracted from the OMOP data model version 4.4 up to 2012. The OMOP project then transitioned to OHDSI [41], which restricted access to such data. Consequently, all information after 2012 has been manually curated from approved drug labels. The current version of DrugCentral indexes 11,775 drug-indication pairs, 2542 drug-off-label use cases, and 27,671 drug-contraindication pairs. The vocabulary (medical concept terms) describing the related diseases and health conditions in DrugCentral has been mapped to SNOMED-CT [42] and UMLS [43]. This further enables extending the mappings to other terminologies, such as disease-ontology [44]. Out of 2497 medical concepts describing drug indications, approximately 65% have been mapped to existing dictionaries and ontologies, as shown in Table 5.

**Table 5:** A summary of medical terms describing drug uses and contraindications

Database	Indications	Contraindications	Off-label uses
DrugCentral Concept ID	2497	1467	860
SNOMED [42] ID	1624	1206	626
UMLS [43] CUI	1612	1196	623
DOID [44]	608	443	293

### FDA adverse event reporting system

DrugCentral indexes FDA FAERS data based on drug names, drug product names, and UNII identifiers [45]. FAERS data not reported by healthcare professionals is discarded, and only ‘suspected drugs’ with more than three reports for each adverse event are considered. Concept names are mapped as MedDRA terms (Medical Dictionary for Regulatory Activities) [46]. The Likelihood Ratio Test (LRT) signal detection procedure [47] is applied to identify drug-MedDRA term combinations with disproportionally high reporting rates. Critical values based on a p-value < 0.05 under the null hypothesis H0 are computed and stored in DrugCentral. H0 compares the reporting rate for a MedDRA term for a drug against the reporting rates for all other MedDRA terms for that same drug. This comparison enables users to post-process the data using other criteria [2]. Significant signals are events with log-likelihood ratios (LLRs) larger than the calculated drug-specific threshold values (LLRT). Since 2020, the procedure has been applied separately for women and men, supporting analyses of sex-based adverse events [3]. The latest update of DrugCentral introduced two age-based groups: FAERS data for pediatric patients (age ≤ 17 years) and geriatric patients (age ≥ 65 years) [4].

According to LRT statistics, 24.3% (81,654), 22.0% (44,375), and 10.8% (22,250) of drug-MedDRA term combinations show strong signals (LLR/LLRT > 5) in neutral, female, and male FAERS data, respectively. In pediatric and geriatric patients, 0.1% (2) and 22% show strong signals, respectively (Table 6). Very strong signals (LLR/LLRT > 10) are provided by 0.7–12.2% of the FAERS data within the sex-based partitioning. Pediatric data are poorly represented in FAERS, whereas 10.5% of the geriatric reports have very strong signals (Table 6).

**Table 6:** Summary of FAERS data (number of drugs in parentheses) according to LRT statistics [47]

LRT thresholds^a^	Sex			Age	
	Neutral	Female	Male	Pediatrics	Geriatrics
LLR/LLRT > 1	335,832 (1957)	202,162 (1655)	134,718 (1517)	170 (82)	258,996 (1806)
LLR/LLRT > 5	816,54 (1302)	44,375 (1042)	22,250 (889)	2 (2)	57,033 (1161)
LLR/LLRT > 10	41,219 (1064)	21,749 (822)	9661 (681)	1 (1)	27,419 (930)

^a^Log-likelihood ratio (LLR), log-likelihood ratio threshold (LLRT)

## Drug products and formulations

**Fig. 6:**
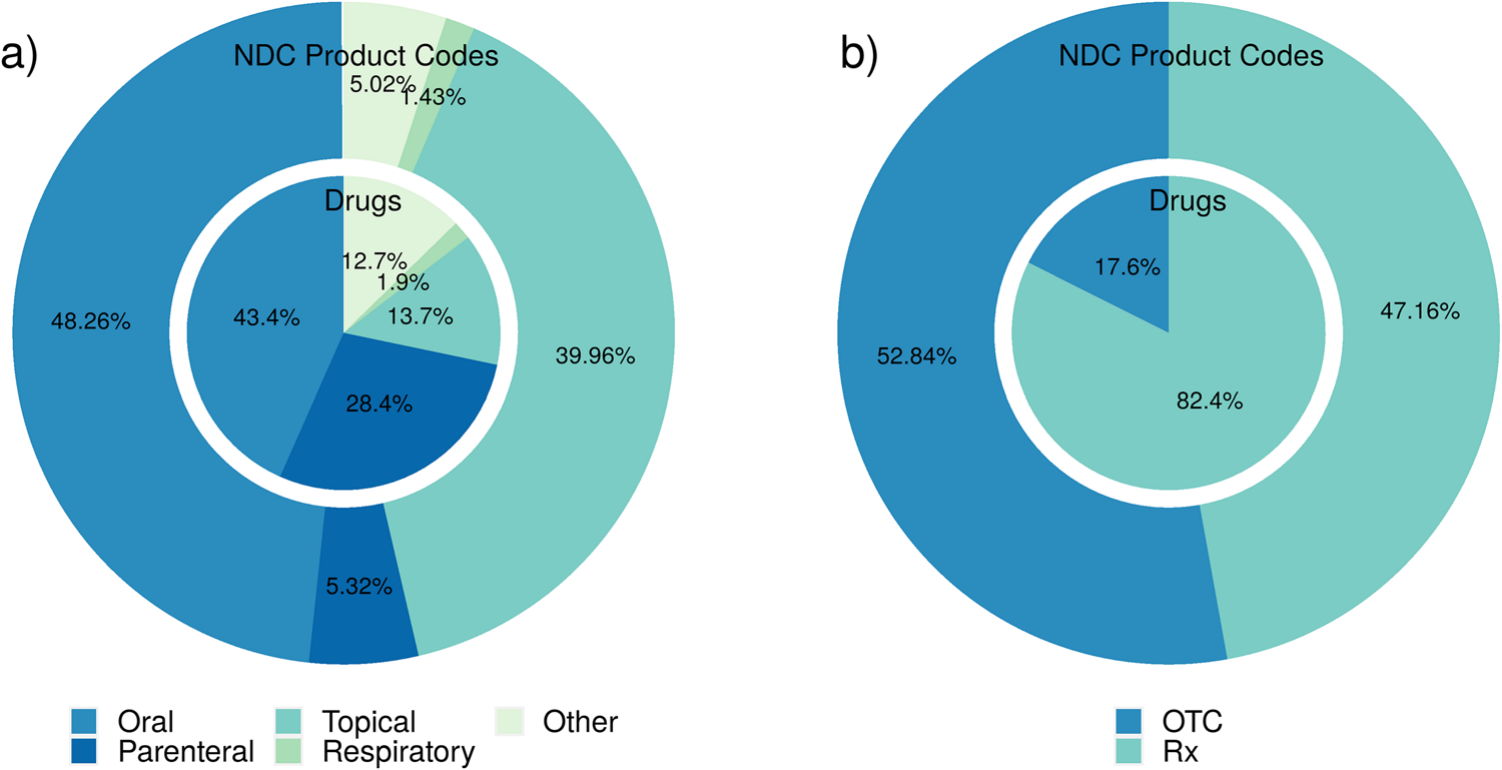
Proportions of administration routes **a** and drug product types **b**; human prescription—Rx, and over-the-counter—OTC products) in FDA drug products (NDC-product codes) and drugs (active drug substances)

Pharmaceutical formulations and drug products marketed (or discontinued) in the US are extracted from DailyMed [48] and the FDA Orange Book [49]. DrugCentral contains a total of 142,303 products and formulations, with most administered orally (48.26%) and topically (39.96%), as shown in Fig. 6a. In terms of drugs (active substances), 1121 (43.4%) are formulated for oral administration, 733 (28.4%) for parenteral, and 354 (13.7%) for topical administration.

Regarding prescription types, 1761 (82.4%) drugs are found in human prescription (Rx) products, and only 375 drugs in over-the-counter (OTCs) products (Fig. 6b). However, the proportion of Rx and OTC products is relatively similar, 67,103 Rx versus 75,200 OTC products. Additionally, full-content drug labels extracted from DailyMed, are stored in DrugCentral and available for query in the text search functionality accessible from the web portal.

## Other features

### L1000 signature

Gene expression changes across multiple cell lines after exposure to drugs and small molecules provided by the LINCS program (Library of Integrated Cellular Signatures) [50, 51] have been integrated into DrugCentral. Gene expression changes induced across 81 cell lines were mapped to 1613 drugs resulting in 8,757,622 drug-cell line combinations made available in DrugCentral. Perturbational similarity across drugs was computed as Pearson correlation. The root mean square deviation (RMSD) and Pearson correlation between the perturbational profiles of the drugs across the cell lines were used to encode similarity. A search interface was developed in R-Shiny and added to DrugCentral to search and browse drugs with the most similar gene perturbation profiles. Correlation/distance profiles for any drug can be queried and downloaded.

### REDIAL-2020

DrugCentral has implemented a web server to support efforts in finding suitable treatments for COVID-19 by quickly and efficiently estimating anti-SARS-CoV-2 activities from molecular structures [52]. The REDIAL-2020 server consists of six machine learning (ML) models representing various experimental assays related to viral entry (VE), viral replication (VR), and live virus infectivity (LVI), extracted from the NCATS (National Center for Advancing Translational Sciences) COVID19 portal (SARS-CoV-2 Assays—NCATS) [53, 54]. The models were built independently using fingerprint, pharmacophore, and physicochemical descriptors, supplied to 22 different ML algorithms from sci-kit-learn [55]. Consensus models were built using the top-ranking model for each descriptor type to maximize prediction performance. The REDIAL-2020 server (http://drugcentral.org/Redial) implements the most predictive models based on extensive performance validation.

## Data access

**Fig. 7:**
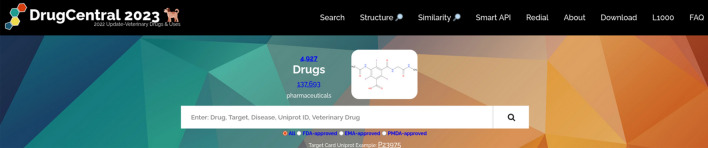
DrugCentral web interface

The DrugCentral web interface (Fig. 7) is accessible at https://drugcentral.org/ from various devices, including desktops, laptops, phones, or tablets. The web search functionality supports multiple search types:

1. Drug search: uses terms such as generic drug names, synonyms, brand names, and identifiers.
2. Target search: supports terms like HUGO gene symbols, UniProt accessions, target names, and Swissprot identifiers.
3. Disease search: can be performed using SNOMED-CT and OMOP vocabulary terms.
4. Pharmacological action search: supports terms like MeSH, Mechanism of Action, physiologic effect (PE), FDA-established pharmacologic class (EPC), and ChEBI action roles [1, 2].

Query results are sorted using a 4-level ranking scheme based on:

A. Drug name or synonyms, mechanism-of-action, and indication terms;
B. Medical terms describing contraindications or off-label uses, targets (not MoA) in bioactivity data, and pharmacological actions;
C. Drug description;
D. FDA drug labels.

The ‘Drugs in the News’ and ‘Featured News’ sections are updated based on recent events [3].

In the DrugCentral web interface, direct access to the ‘Redial’ and ‘L1000 signature’ portals is available. As a result, users can estimate anti-SARS-CoV-2 activities of chemical structures based on the REDIAL-2020 models [3], and browse the distance/correlation matrices of the L1000 ConnectivityMap perturbation profiles [2]. By clicking the ‘About’ button on DrugCentral’s web page, users can access a summary of the database, including charts and functional tables that illustrate the current content of the database.

The full DrugCentral database is available for download in PostgreSQL format, enabling advanced data query, export, and integration. Users can load the full database dump file into a local PostgreSQL instance and perform advanced data manipulation using multiple structured query language (SQL) examples available in the download section of the DrugCentral website [1]. Several user-requested downloads are also made available, including chemical structures of the drugs in structure-data files (SDF), SMILES, and InChI formats, and drug bioactivity profiles in tabular format [2]. These resources allow users to access and work with DrugCentral data more flexibly and efficiently, depending on their specific needs and preferences.

## Web-interface use case: warfarin

DrugCentral can be browsed online through the text search functionalities implemented in the web interface. As an example, let’s consider a drug search for warfarin.

Warfarin, a widely-used anticoagulant, is primarily prescribed to prevent blood clots from forming and growing in people with various conditions such as heart attack, certain types of irregular heartbeat, prosthetic heart valve implants, and to treat or prevent venous thrombosis and pulmonary embolism. Searching DrugCentral for the drug name lists warfarin first, followed by several pharmacologically similar anticoagulants based on the sorting criteria (see the above section for sorting criteria).

The drug card view for warfarin contains several tables with information about the drug class, dosage, pharmacokinetic properties, regulatory information, adverse events, pharmacologic actions, indications, bioactivity profile and MoA targets, external identifiers, and pharmaceutical formulations. Briefly, the description tab includes:

1. The chemical structure of warfarin (downloadable in several formats).
2. A list of synonyms.
3. A short description extracted from the drug label.
4. Calculated physicochemical properties.
5. The drug’s status (Off Patent) shows that, while currently marketed, warfarin has expired composition-of-matter patents (see US patent US3077481A). This classification can help prioritize compounds in drug repositioning studies.

**Fig. 8:**
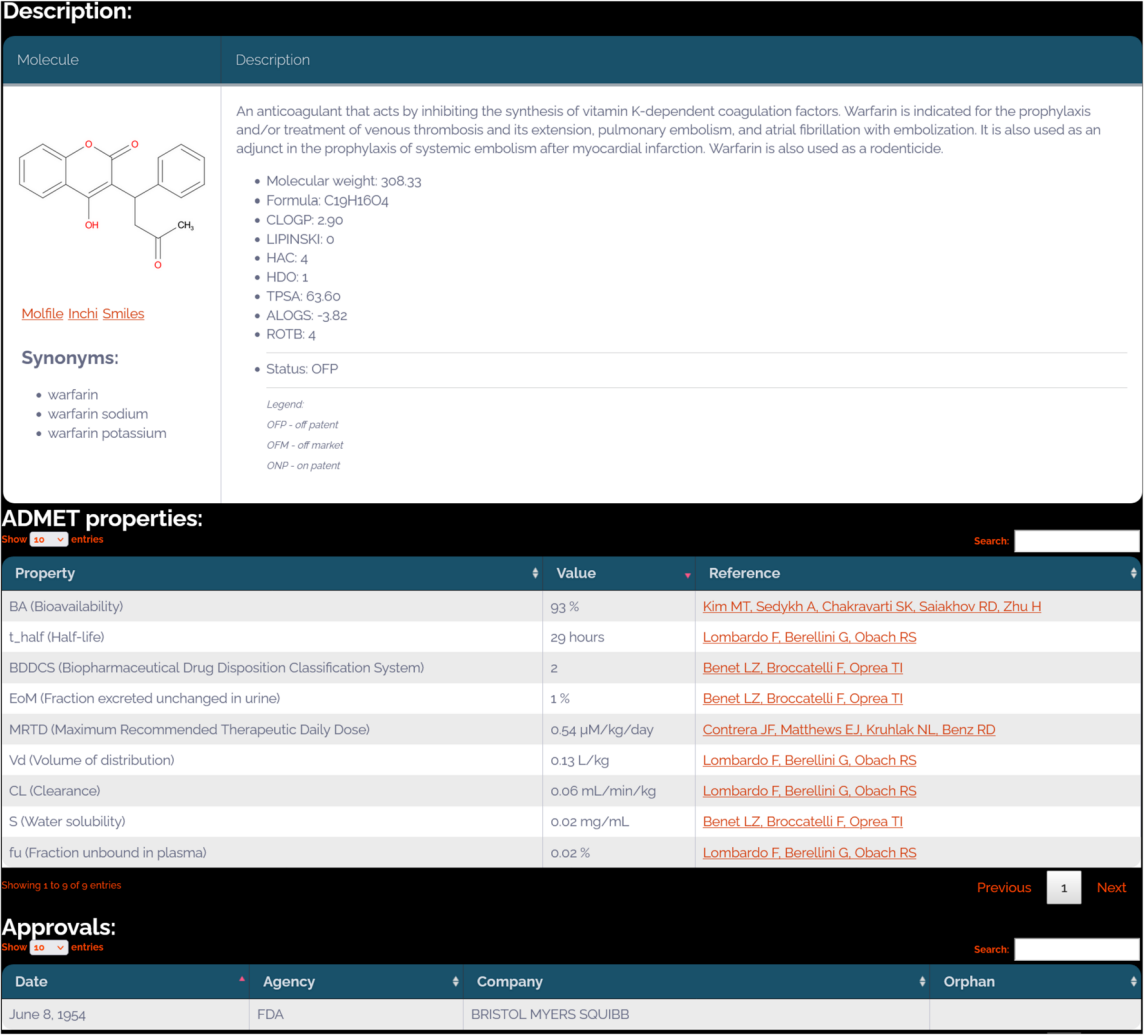
Warfarin search results in DrugCentral: description, ADMET properties, and approval panels

Nine pharmacokinetic parameters (manually curated from the scientific literature) are shown for Warfarin in the ADMET properties table. The Approvals table includes the first approval date of Warfarin (June 8, 1954) and other regulatory information. See Fig. 8.

**Fig. 9:**
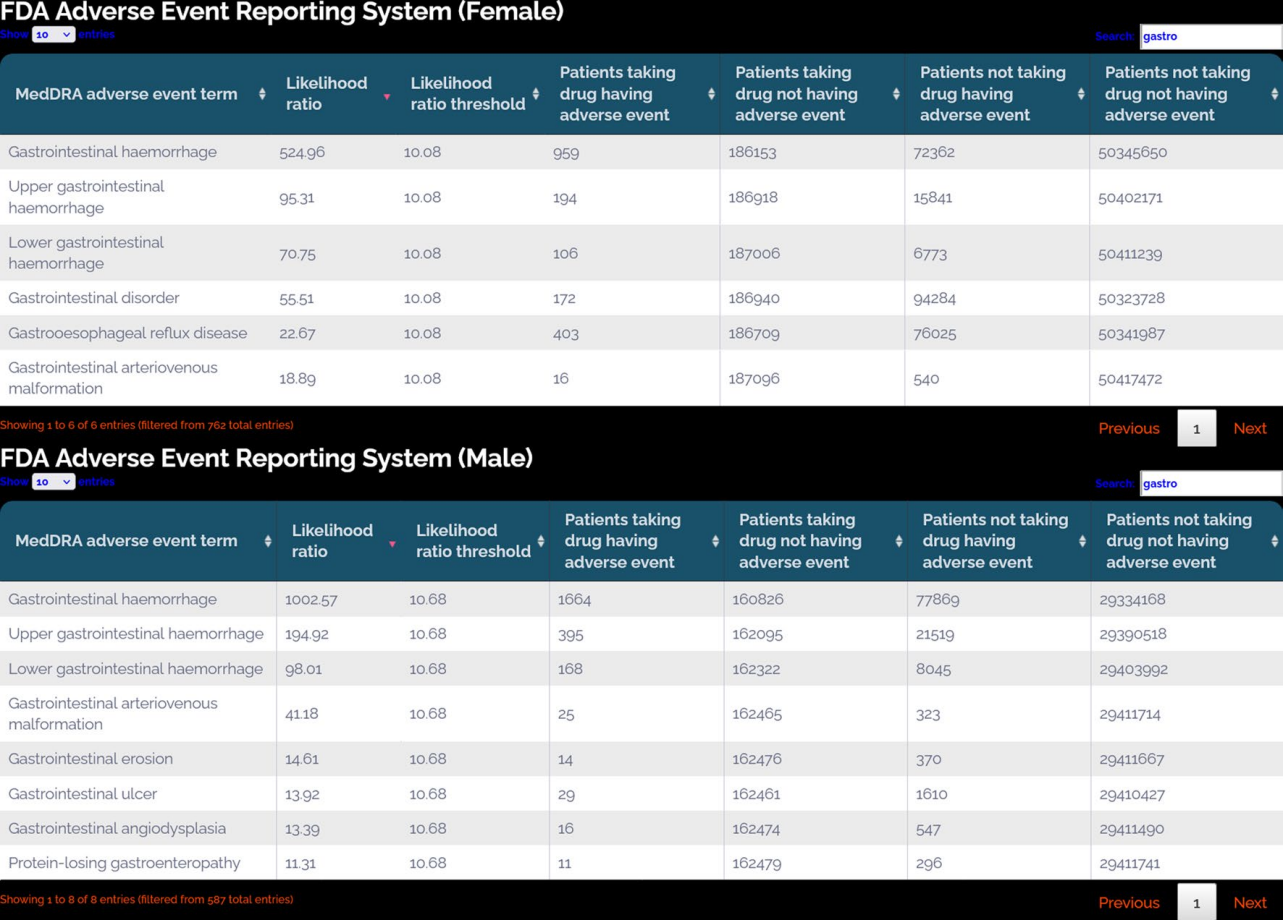
Adverse effects of warfarin separated by sex

DrugCentral provides information about FDA post-market adverse drug events for Warfarin, separated by sex (Fig. 9). Significant signals can be encountered for adverse effects with log-likelihood larger than the threshold values. For warfarin, gastrointestinal hemorrhage is frequently encountered in both men and women but has a significantly higher (almost double) occurrence in males (Fig. 9). Reports of a higher risk of gastrointestinal bleeding in men versus women under treatment with warfarin [56] confirm the results from DrugCentral. Such information can be used by healthcare professionals to assess the risks associated with Warfarin treatment and adjust the treatment plan accordingly for different patient populations [56].

**Fig. 10:**
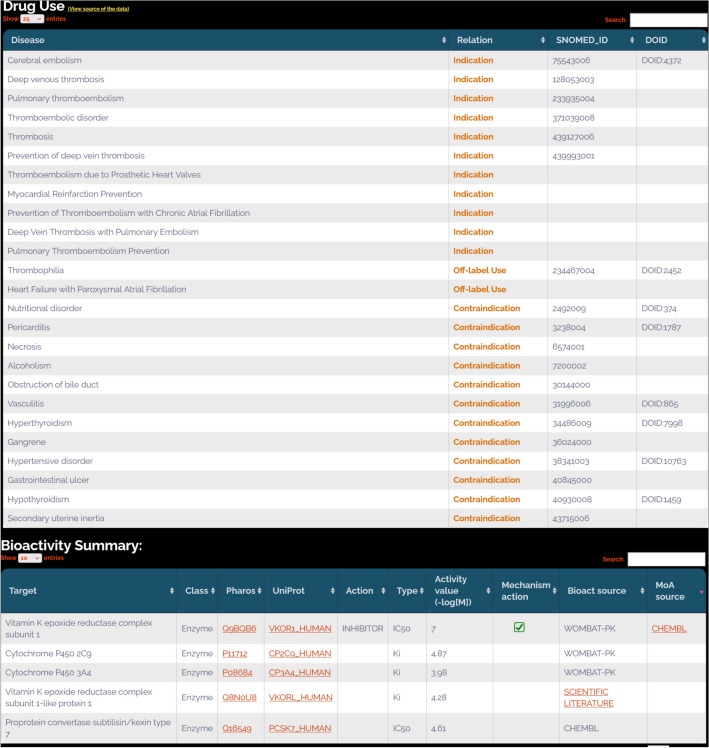
Warfarin search results in DrugCentral: drug use and bioactivity panels

The therapeutic uses of warfarin cover various sorts of venous thromboembolisms (embolisms due to prosthetic heart valves) and atrial fibrillation (Fig. 10). Off-label uses and contraindications are also provided in the Drug Use panel. Indications and contraindications for Warfarin are extracted from drug labels and linked to clinical terminology dictionaries, such as SNOMED-CT and Disease-Ontology. DrugCentral enables efficient information retrieval and seamless integration with other medical data sources by mapping these terms to standardized vocabularies. This information is crucial for healthcare providers to make informed decisions when prescribing Warfarin, as it provides an overview of the approved and non-approved treatment scenarios where the drug can be used or should be avoided.

The bioactivity profile of warfarin is captured in the Bioactivity summary panel (Fig. 10). The target names are provided with links to Pharos and Uniprot databases, offering users easy access to more in-depth information about the target proteins. A checkbox annotates Vitamin K epoxide reductase complex subunit 1 (VKORC1) as the MoA target for Warfarin, based on information from ChEMBL. By enabling such user-friendly access, DrugCentral provides a comprehensive overview of drug-protein interactions. Such information is essential for understanding the molecular basis of therapeutic and off-target effects, which can be relevant for drug repurposing efforts or developing new therapeutic agents with similar mechanisms of action.

## Drug repurposing use-case: pharmacokinetics considerations

Drug repurposing projects aim to discover new therapeutic applications for existing drugs. Over the past few years, there has been a significant increase in drug repurposing studies [57, 58], which include various computational approaches [59, 60]. For example, docking and molecular dynamics (MD) studies of ~ 2000 drugs against the main protease (Mpro) of SARS-CoV-2 led to the identification of 5 drugs (manidipine, boceprevir, lercanidipine, bedaquiline, and efonidipine) with IC_50_ values between 4.8 and 38.5 μM [61]. Such concentrations are relatively high in comparison to the drug plasma concentration, e.g., oral administration of 10 mg lercanidipine results in a Cmax (the highest concentration in the blood) of 0.015 uM (~ 9.2 ng/mL) [62], raising concerns regarding the true repurposing potential of these drugs to treat COVID-19. Such studies have been criticized for not taking into account essential drug development factors, such as pharmacokinetics [57, 63]. In this use-case example, we will use the data available in DrugCentral to establish an activity threshold based on pharmacokinetic parameters, which can help guide drug repurposing efforts.

Out of 1156 drugs with defined MoA human targets and available activity values, 822 are categorized as OFP/OFM/ONP, as described in Table 7. The activity values for these targets (expressed as − log[ACT/M], further denoted as pACT) are the highest for ONP with a mean of 8.30 and decrease to 7.62 for OFP and to 7.42 for OFM drugs. This indicates that more recently approved drugs tend to have increased potency (Table 7). Moreover, ONP drugs tend to show different pharmacokinetic properties as encoded by the Biopharmaceutics Drug Disposition Classification System (BDDCS) parameter. BDDCS classifies drugs into four categories according to water solubility and extent of metabolism, EoM: Class 1—high solubility and high EoM; Class 2—low solubility and high EoM; Class 3—high solubility and low EoM; and Class 4—low solubility and low EoM, respectively [15, 64]. BDDCS properties are also driven by the subcellular localization of the biomolecular drug targets [65].

**Table 7:** Brief description of the repurposing categories (OFP, OFM, ONP) for drugs with defined MoA targets

Class	No. of drugs	pACT^a^	
		Mean/median/sd	80%
OFP	537	7.62/7.80/1.55	> 6.22
OFM	99	7.42/7.54/1.59	> 5.56
ONP	186	8.30/8.33/1.09	> 7.48

^a^− log[ACT/M]

**Fig. 11:**
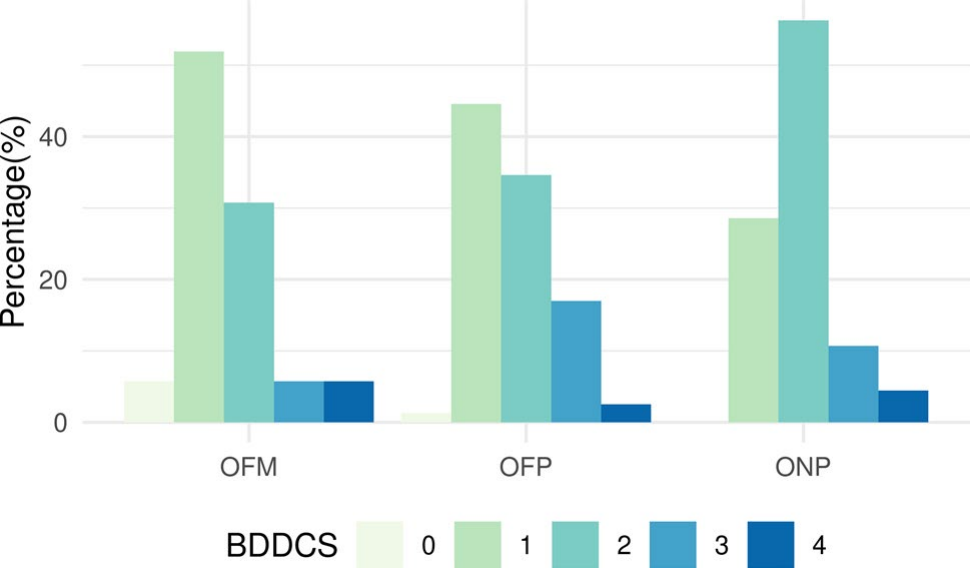
Percentages of BDDCS classes in off-market (OFM), off-patent (OFP), and on-patent (ONP) drugs

DrugCentral BDDCS annotations were updated with data from Bocci et al. [15], with 342 novel BDDCS-drug annotations and 82 updates. Out of a total of 1391 drugs with BDDCS, 715 have MoA targets. Of these, 81% are classified as BDDCS 1 and 2. OFP and OFM drugs are predominantly BDDCS 1 (45 and 52%, respectively), i.e., high solubility, whereas 56% of ONP drugs are BDDCS class 2, i.e., low solubility and extensive metabolism (Fig. 11). Since water solubility data is captured in DrugCentral we further investigated its influence on target bioactivity.

Most drugs (87%) show pACT > 6 on human MoA targets, a reasonable activity threshold (t_pACT_) a drug should exceed against a potential MoA target for drug repurposing. DrugCentral currently contains 1146 drugs with solubility information. Of these, 468 are drugs with MoA pACT > 6. For illustrative purposes, we examined only the lowest bioactivity value (the lowest MoA potency when multiple targets were annotated), and converted solubility values to logS. The Δ = log(S/ACT) computation shows the log difference between bioactivity and solubility. For 84% of the cases, Δ > 3. For any drug *D* we can define *t*_*pACT_sol_D*_ = 3 − logS_D_. In other words, *for a drug to be successfully repurposed, its activity against a novel (repurposed) target should be at least 3* − *logS*.

Poorly soluble drugs (lower logS) would require higher pACT values compared to highly soluble drugs with lower *t*_*pACT_D*_. This is consistent with our earlier observation that ONP drugs are, on average, more potent and less water-soluble than OFP and OFM drugs. Given the activity threshold introduced earlier (*t*_*pACT*_ of 6), the “3 − logS” is more useful for drugs with logS < − 3. Thus, a novel drug-target activity could be considered “viable” for repurposing when *t*_*pACT*_ = *t*_*pACT_sol*_ if *t*_*pACT_sol*_ > 6 and logS < − 3. We caution that this rule is derived from a limited dataset and ignores other factors critical for drug repurposing candidates [57, 63], and should be considered in the appropriate context. Further validation is required to confirm its applicability in drug repurposing.

Water solubility for warfarin is 0.02 mg/mL, i.e., logS of − 4.23 (logS < − 3), which results in a *t*_*pACT_sol*_ of 7.23 (0.06 µM). This value can serve as a *de minimis* bioactivity (*t*_*pACT*_) guideline, should one consider warfarin as a repurposing candidate. BDDCS in general, and water solubility in particular, highlight the importance of considering pharmacokinetics information when repurposing drugs. The argument for prescribing warfarin for an unmet medical condition has to outweigh its potentially severe side effects given its on-target medical use (anticoagulant). Thus, scientists should consider the intended on-target effects and safety profile before proposing repurposing candidates.

## Data Availability

DrugCentral database is available at https://drugcentral.org/, free of charge, without registration.

## Abbreviations

MoA: Mechanism-of-action
FDA: U.S. food and drug administration
EMA: European medicines agency
PMDA: Japan pharmaceutical and medical devices agency
ADMET: Absorption, distribution, metabolism, excretion, and toxicity
FAERS: FDA’s adverse event reporting system
MeSH: Medical subject headings
mAb: Monoclonal antibodies
GPCRs: G-protein coupled receptors
