# Correction: Exploring DrugCentral: from molecular structures to clinical effects

**DOI:** 10.1007/s10822-023-00545-x

**Published:** 2023-12-02

**Authors:** Liliana Halip, Sorin Avram, Ramona Curpan, Ana Borota, Alina Bora, Cristian Bologa, Tudor I. Oprea

**Affiliations:** 1 Department of Computational Chemistry, “Coriolan Dragulescu” Institute of Chemistry, Timisoara, Romania; 2 Translational Informatics Division, Department of Internal Medicine, University of New Mexico School of Medicine, Albuquerque, NM USA; 3 Expert Systems Inc, San Diego, CA USA

**Correction to: Journal of Computer-Aided Molecular Design (2023) 37:681–694**

10.1007/s10822-023-00529-x

The article “Exploring DrugCentral: from molecular structures to clinical effects”, written by Liliana Halip, Sorin Avram, Ramona Curpan, Ana Borota, Alina Bora, Cristian Bologa, Tudor I. Oprea was originally published Online First without Open Access. After publication in volume 37, issue 12, page 681–694 the author decided to opt for Open Choice and to make the article an Open Access publication. Therefore, the copyright of the article has been changed to © ©The Author(s) 2023 and the article is forthwith distributed under the terms of the Creative Commons Attribution,

This article is licensed under a Creative Commons Attribution 4.0 International License, which permits use, sharing, adaptation, distribution and reproduction in any medium or format, as long as you give appropriate credit to the original author(s) and the source, provide a link to the Creative Commons licence, and indicate if changes were made. The images or other third party material in this article are included in the article’s Creative Commons licence, unless indicated otherwise in a credit line to the material. If material is not included in the article’s Creative Commons licence and your intended use is not permitted by statutory regulation or exceeds the permitted use, you will need to obtain permission directly from the copyright holder. To view a copy of this licence, visit http://creativecommons.org/licenses/by/4.0.

The original article has been updated.

